# Quality of care in family planning services in Senegal and their outcomes

**DOI:** 10.1186/s12913-017-2287-z

**Published:** 2017-05-12

**Authors:** Shireen Assaf, Wenjuan Wang, Lindsay Mallick

**Affiliations:** 1 ICF, The Demographic and Health Surveys (DHS) Program, 530 Gaither Road, Suite 500, Rockville, MD 20850 USA; 2 Avenir Health, The Demographic and Health Surveys (DHS) Program, 530 Gaither Road, Suite 500, Rockville, MD 20850 USA

**Keywords:** Quality of care, Health facilities, SPA, Counseling, Family planning, Client satisfaction, Knowledge of method protection from STIs, Senegal

## Abstract

**Background:**

High quality of care in family planning (FP) services has been found to be associated with increased and continued use of contraceptive methods. The interpersonal skills and technical competence of the provider is one of the main components of quality of care. To study the process component of quality of care, the distribution of the FP counseling topics was examined by client, provider and facility characteristics. To assess the outcomes of quality of care, client satisfaction and their knowledge of their method’s protection from STIs were used. This study examined the factors associated with these outcomes with a focus on provider counseling and training.

**Methods:**

Data from the 2012–2013 Senegal Service Provision Assessment survey was used for the analysis. The survey included a representative sample of the health facilities in Senegal and collects data by observing the clients’ FP visits and conducting exit interviews. The main outcomes of interest were provider’s counseling in FP, client’s satisfaction with FP services and client’s knowledge of their method’s protection from STIs. Several covariates were used in the analysis which represent client, provider and facility characteristics.

**Results:**

The level of counseling was inadequate-- very low proportions of providers that performed different types of counseling. Counseling was more likely to be provided to new than returning clients. Approximately 84% of the clients were very satisfied with services but only 58% had correct knowledge of their method’s protection from STIs. Clients were significantly less likely to be very satisfied when their providers counseled on side effects and when to return, and counseling provided on method’s protection from STIs did not significantly improve knowledge in this area. Clients seen by a provider with FP training had almost twice the odds of having correct knowledge about their method’s protection from STIs compared with clients seen by a provider with no recent training.

**Conclusions:**

The percentage of providers offering FP counseling to their clients was relatively low and was ineffective on the client-focused outcomes. Interventions may be required for more effective counseling methods that are client-centered as well as providing more FP training to providers.

## Background

While fertility declines have been observed globally, fertility in sub-Saharan Africa has declined at a slower pace in comparison [1]. The fertility rate in sub-Saharan Africa was estimated at 5.1 births per woman in the 2010–2015 period and projected to be 4.75 in 2015–2020 [2]. In comparison, the fertility rates in the same periods for Asia and Latin America and the Caribbean are reaching very near replacement levels [2]. One of the pathways to fertility decline is the use of family planning methods, especially modern contraception; however, a high proportion of women still have an unmet need for contraception [1]. In Senegal, while the percentage of women in union who use a modern contraceptive method has increased over the years, it still remains relatively low at 20% in 2014 [3]. Total fertility rates remain high at 5.0 children per woman, with almost half of the population under 15 years, and about 30% of married women have unmet need for family planning as estimated by the Demographic and Health Survey (DHS) in 2014 [3]. Unmet need for contraception can be due to several obstacles including, but not limited to, lack of knowledge of contraceptive methods, concerns of side effects, stockouts, as well as low quality family planning services [1]. A multi-country analysis based on Demographic and Health Survey (DHS) data in 15 countries showed that between 7–27% of women stopped using contraception because of a reason related to quality of care [4]. High quality of care in family planning services has been found to be associated with increased and continued contraceptive use in several settings [4–10]. In a longitudinal analysis in Bangladesh, Koenig et al. found women had a 60% higher likelihood of adopting a modern method and a one-third lower likelihood of discontinuing the method if they received a higher quality of family planning services from fieldworkers [7]. A panel study conducted in the Philippines also showed the importance of the quality of initial family planning services women received - continuation of use significantly increased with quality [11] Research in Senegal has also found that women who received good quality of care in health facilities were more likely to use a contraceptive method compared to others [12]. More specifically, providing counseling to clients during family planning services was found to improve both long term outcomes, such as increased birth spacing and continued use of modern contraception methods, as well as short term outcomes such as increased knowledge and satisfaction with family planning services [12–15]. A study in Egypt has also highlighted the importance of client-centered counselling to improve outcomes [13].

Measuring quality of care can be challenging as it can have many different definitions and components. Donabedian [16] discusses the measurement of quality of care in three components: structure (including infrastructure of the health facility, equipment, and its commodities), process (the health provider’s method of delivering care including interpersonal interaction with the client and technical competency), and outcome (the outcome of the service provided such as their satisfaction with services). Bruce [17] provides a framework for examining quality of care that is specific to family planning which focuses on the process and outcome components discussed by Donabedian [16]. The framework covered six components namely choice of methods, information given to clients, technical competence of providers, provider-client interpersonal relations, mechanisms for encouraging continuity and follow up and appropriate constellation of services [17]. Bruce recommended examining quality of care in family planning services by using the outcomes of client knowledge, client satisfaction with services, and contraceptive use behavior. Many of the indicators required to study these aspects of quality of care in family planning can be obtained by either interviewing clients visiting the facilities or through observing interactions between the provider and the client. However, Bessinger and Bertrand suggested a combination of observations and clients exit interviews for a better assessment of quality of care in family planning facilities [18].

The focus of this analysis was to examine the provider-related process and the client-focused outcomes components of quality of care and specifically the effect of process on the outcomes of client satisfaction and their knowledge of their methods protection from STIs. The analysis originated from an earlier study performed by the same authors [19] with a focus on the process and outcomes of quality of care. This analysis will examine the counseling provided in family planning facilities in Senegal to assess the level as well as the distribution of the counseling provided by client, provider and facility characteristics. In addition, the paper studies the effect of counseling on the client’s satisfaction with services and their knowledge of contraceptive methods. The effect of other variables on client’s outcomes will also be examined which include other provider related variables such as their training, as well as client and facility variables. As Senegal has made fertility reduction one of its priority areas in the National Health Development Program (PNDS) for 2009–2018 [20], the findings from this study can help to inform policy interventions aimed at increasing the quality of care in family planning services for the purpose of improving long and short term family planning outcomes.

## Methods

### Data

The data used in this analysis was from the Senegal Service Provision Assessment (SPA) survey conducted in 2012–2013. This survey is part of the Senegal Continuous Survey project which contains a SPA component and a DHS component; it is designed to have five annual rounds of data collection, with the last round in 2017. The SPA surveys provide comprehensive and detailed information on how the country delivers health services. They are conducted by taking a national sample of formal health care facilities in the country. Each round covers a representative sample of facilities, including 50% of all hospitals and health centers in Senegal and 20% of all health posts and a sample of their associated health huts [21]. The health care system in Senegal is organized in a pyramid design, with hospitals representing the top of the pyramid, followed by health centers, and finally health posts and huts at the lower levels [22].

SPA surveys are conducted using four instruments: 1) health facility inventory, 2) health provider interview, 3) observation checklist, and 4) client exit interview. The analysis uses data from all instruments but mainly from the observation and client exit interviews. The health facility inventory includes information about the components of service that are necessary for providing priority care: infrastructure, equipment, commodities, and medicines. For the health provider interview, health workers were selected and interviewed with a questionnaire that covers information on providers’ qualifications, training, and the type of services they provide. Consent for conducting the interviews and observations was obtained from the facility, provider and client. The consent was read by the interviewer and signed by the interviewee.

All rounds of the Senegal SPA use the same inventory and health provider questionnaires. In the first round (2012–2013 survey), the observation and exit interviews were conducted for family planning and child curative services and therefore the data from this round was used in the analysis. The observation checklist examined adherence to international protocols and the level of counseling provided. At the end of the observation, the clients were interviewed to assess their level of satisfaction with the services as well as their knowledge. Health huts did not have observation checklists or exit interviews and therefore were not included in the analysis. In addition, based on the 2012–13 Senegal DHS, approximately 2% of women reported health huts as their source of contraceptive methods.

A total of 364 facilities (excluding health huts) were included in the survey from which 968 family planning consultations were observed. Providers whose consultations were observed were automatically selected for the health worker interview. In addition, all family planning clients whose consultations were observed were eligible for the exit interview. This analysis focuses on clients that were either prescribed or provided a modern contraceptive method during the observed visit. This provided a weighted sample of 872 family planning consultations for the analysis of the process and outcome components of quality of care.

### Analysis

Data for the analysis of the provider-related process component of quality of care came from direct observations of family planning consultations and physical examinations. The process assessment focused on the quality of counseling. There was only one male in the observation sample; therefore, the analysis was restricted to female clients who were provided or prescribed a method during the observed visit. The quality of counseling was measured with four binary indicators on the content of information exchanged between the provider and the client during the counseling session: whether the provider counseled on how to use the method; whether the provider discussed side effects of the method; whether the provider gave advice on when to return for follow-up services; and whether the provider counseled about method protection from STIs. These indicators were defined specifically for the method provided or prescribed to the client (Table 1). To assess the overall quality of counseling, a binary variable was also created for all counseling methods used, based on whether the client was counseled on all three aspects about the method: how to use it, side effects, and when to return to the facility.

**Table 1:** Family planning process variables: Questions used for constructing indicators on quality of counseling

Method	How to use^a^	Side effects^b^	When to return^b^	Protection from STIs^a^
Pills or injectables	When to take	Initial side effects that may occur (such as nausea, weight gain, and breast tenderness)	Return to clinic if side effect appears	Method does not protect against STIs, including HIV
	What to do if forget			
Condoms	Each can be only used once			Dual protection
Intrauterine device (IUD)	Good for up to 5–12 years	Common side effects that may occur	Return to clinic 3–6 weeks post-insertion or after first menses	Method does not protect against STIs, including HIV
	Users should regularly check strings after each menstruation		Return to clinic if side effects continue	
Implants	Good for 3–5 years	Initial side effects that may occur (such as nausea, weight gain, breast tenderness)	Return to clinic if side effects continue	Method does not protect against STIs, including HIV
Periodic abstinence or standard days method (SDM)	How to identify a woman’s fertile period			Method does not protect against STIs, including HIV
	No intercourse during woman’s fertile period without alternative method (condom)			
Lactational amenorrhea method (LAM)	Must be exclusively (or near-exclusively) breastfeeding			Method does not protect against STIs, including HIV
	Not effective after menstruation begins again			
	Infant must be less than age 6 months			

Only methods provided or prescribed to observe clients were included

^a^Apply to users of all methods reported

^b^Apply to only users of pills, injectables, IUDs, and implants

Two dependent variables were used to study the client-focused outcome component of quality of care in family planning. The first variable used a question in the family planning client exit interview that asks clients about their overall satisfaction with the services provided. The categories for response were: (1) very satisfied, (2) more or less satisfied, and (3) not satisfied. Based on the distribution of these responses, a binary dependent variable was created in which the response “more or less satisfied” was combined with “not satisfied.” This grouping was performed due to the high level of satisfaction found in Senegal. Grouping the “more or less satisfied” category (15.7%) with “very satisfied” (83.7%) would have created very little variability in the outcome as only 0.6% were found to be not satisfied with services. As discussed by Donabedian [11], the low level of reported dissatisfaction could be partially due to client’s feeling they do not want to speak against their providers. Therefore, this analysis will examine respondents that are very satisfied with their services. The second dependent variable used to study the outcome component, also a binary variable, indicates the knowledge of the client after receiving services, and was derived from a question asked during the family planning exit interview. The question asks the client if their method protects them from STIs, including HIV/AIDS. The method the client was using was determined from the observation of the family planning consultation, during which the method or methods provided or prescribed to the client were recorded. Clients who answered yes and who were not using condoms were coded as having incorrect knowledge, those who answered no were coded as having correct knowledge (unless they were using condoms), and those who answered that they did not know were removed from the analysis. Approximately 16% of clients did not know whether the method they were using protects from STIs (results not shown). Further analysis of the respondents in the “do not know” category does not clearly indicate whether to group them with the respondents who answered either “yes” or “no,” as we cannot know for certain how they would have answered if probed further. This further analysis included using a multinomial logistic regression of this outcome with three categories of do not know, correct knowledge, and incorrect knowledge. The results indicated that respondents from the “do not know” category appeared to behave more similarly to the respondents that had correct knowledge category. Since we could not determine with certainty where to group the respondents from the “do not know” category, these respondents were removed from the analysis. Consequently, the denominator for this variable differs from that of the satisfaction outcome. The analysis of client satisfaction was restricted to users of pills, injectables, IUDs, and implants (all users except for 10 cases), and the analysis of the knowledge outcome was restricted to all users. This was because two variables on counseling required in the satisfaction outcome analysis (counseling on side effects of method and when to return) were only available for users of pills, injectables, IUDs, and implants. All the outcome analyses on quality of care were restricted to female clients.

The independent variables used in the analysis are divided into three groups: the background characteristics of the client, the provider, and the facility. The relevant independent variables were selected for each process and outcome dependent variable. The provider’s characteristics were drawn from the health provider interview. The client’s characteristics were taken mainly from the client exit interviews, except for client status (new or returning client), family planning method currently being used, and whether the client left the clinic with a method, which were drawn from the observation of the family planning consultation.

For client’s age, 52 clients who responded that they do not know their age were placed with the oldest group, age 40–58, on the assumption that the oldest were least likely to know their age. Similarly, 21 clients who responded that they did not know how long they waited to see a provider were placed with the largest waiting category of two hours or more, on the assumption that clients who had to wait very long might not remember exactly how long they waited. As for the provider characteristics, only four specialists and general physicians were observed, and these were placed within the nurse or nurse assistant category. One important characteristic was supervision that the provider received during the six months before the survey. This refers to technical support or supervision from a facility-based supervisor or from a visiting supervisor in various forms, including review of records or reports, observation of work, feedback on work performance, and discussion of problems encountered by the provider. A three-category variable was constructed to measure supervision: received none, received one to five supervisory items, and received all six listed supervisory items.

For the provider-related process analysis, the independent variables included the client’s background characteristics (age, education, new or returning, and contraceptive method currently being used by the client), the provider’s background characteristics (provider category, years of education, training received, supervision, having a job description, and salary type), and the facility characteristics (health facility type, locality, region, and the equipment composite index). Years of education received was used as a proxy for the number of years of experience (Choudhry, Fletcher, and Soumerai 2005). In addition, the variable salary type was included as a measure that might influence the provider’s motivation, which in turn could affect their performance (Rowe et al. 2005). The facility characteristics, and specifically the equipment composite index, represent the structure component, which was discussed by Basinski et al. and Donabedian (Basinski et al. 1992; Donabedian 1988) as possible predictors of the process component of quality of care. While the presence of equipment in a facility may not necessarily have a direct effect on the process or the provider’s level of care, it may serve as a proxy for the overall environment and readiness of the facility, and is included as a predictor of the process quality of care for this purpose. This structure equipment index was created by PCA method from a number of minimum required equipment in a facility as defined by the WHO SARA guidelines [23].

The analysis of client’s overall satisfaction included the client variables of fee paid for service, waiting time, and whether the client left with a family planning method (Agha and Do 2009; Aldana, Piechulek, and Al-Sabir 2001; Hutchinson, Do, and Agha 2011). In addition, the process dependent variables were included as covariates. These included variables based on providing counseling on how to use the method, the side effects of the method, and when to return if side effects appeared. Counseling on side effects and counseling on when to return were highly correlated with each other and were not included in the same regression model. Therefore, two models were constructed for the satisfaction outcome, Model I with the counseling on side effects covariate and Model II for the counseling on when to return covariate. These covariates were only asked of user of pills, injectables, IUDs, and implants and not all users (difference of only 10 cases). The facility characteristics and the equipment composite index were also added as covariates in the outcome analysis.

Finally, for the analysis of knowledge of the method’s protection from STIs, a variable representing whether the provider provided counseling on this issue was included as a covariate. The client variables used were age, education, status and the contraceptive method that was currently being used by the client.

All analyses took into consideration the stratified sample design and the client’s sampling weight.

## Results

### Description of Study Population

Table 2 describes the independent variables used in the process and outcome analysis for users of modern family planning methods (a total of over 90% of all clients interviewed were either prescribed or provided with a modern method). Most of these clients were age 25–39 (58%), had no education (49%), were returning clients (69%), and were mainly using progestin-only injections for family planning (60%). Almost all the clients left the facility with a family planning method (94%).

**Table 2:** Description of client, provider, and facility background characteristics after selecting for clients that are either prescribed or provided a contraceptive method

Variable	Category	% (Weighted N)
Client characteristics		
Client’s age	14–24	26.6 (232)
	25–39	57.6 (502)
	40–58 and don’t know	15.8 (138)
Client’s education	No education	49.3 (430)
	Primary and post-primary	30.3 (264)
	Secondary or more	20.4 (178)
Fee paid for service^a^	0–9	7.7 (67)
	10–499	14.4 (125)
	500–999	46.6 (407)
	1,000 or more	31.3 (273)
Waiting time	No wait	12.8 (111)
	Less than half hour	29.4 (257)
	Half hour to one hour	19.8 (173)
	One hour to 2 hours	18.4 (161)
	2 hours or more	19.6 (171)
Client status	New client	30.7 (268)
	Returning client	69.3 (605)
Contraceptive method used	Pills	24.8 (216)
	Progestin-only injection	60.3 (526)
	IUD or implants^b^	14.9 (130)
Client left with a method	Yes	94.0 (820)
	No	6.0 (52)
Provider characteristics		
Provider category	Nurse or nurse assistant^c^	32.2 (281)
	Midwife and other	67.8 (592)
Provider sex	Male	18.2 (159)
	Female	81.8 (713)
Provider years of education	6–12	6.5 (56)
	13–16	58.2 (507)
	17+	35.4 (309)
Provider training in family planning in the past 24 months	Yes	45.1 (393)
	No	54.9 (479)
Provider activities supervised	None	20.7 (181)
	1–5	28.8 (251)
	6	50.4 (440)
Provider has a job description	Yes	57.8 (504)
	No	42.2 (368)
Provider salary type	Monthly or daily salary	63.7 (556)
	No regular salary but other compensation	26.0 (227)
	None	10.2 (89)
Health facility characteristics		
Health facility type	Hospital/health center	19.0 (166)
	Health post	81.0 (706)
Locality	Urban	52.1 (454)
	Rural	47.9 (418)
Region	Northern	18.0 (157)
	Dakar	27.6 (241)
	Thiès	14.4 (126)
	Central	19.4 (169)
	East	4.4 (38)
	South	16.2 (142)
General structure equipment composite index	Low	40.5 (353)
	Medium	27.0 (235)
	High	32.6 (284)

^a^Currency in CFA, 1 USD ~ 600 CFA

^b^Includes 10 respondents who used other methods, which were male condoms, LAM, and counseling on periodic abstinence

^c^Includes 4 unweighted doctors and specialists for users

One-third of family planning providers (32%) were nurses or nurse assistants, while two-thirds (68%) were midwives or other providers. (Only 41 of the 592 providers in this group were classified as other.) Correspondingly, 82% of the providers were female since it is unusual for men to be midwives. Most providers had 13–16 years of education (58%) and just over half (55%) had not received any family planning training in the past two years.

As for the facility characteristics, hospitals and health centers were combined in the analysis due to the small number of observations and exit interviews conducted in hospitals. The majority of these interviews were conducted in health posts (81%), and in urban areas (52%), and 28% were in Dakar.

### Family Planning Provider-Related Process

Table 3 shows the quality of counseling by selected characteristics of clients, providers, and facilities. Overall, the quality of counseling was poor. Just 18% of clients were counseled on all three important aspects of their method: how to use the method, possible side effects, and when to return to the facility. Among 872 observed female clients who were provided or prescribed a method, fewer than two-thirds (63%) received information on how to use the method, such as dosage and frequency of use, duration of effectiveness, and correct use of natural family planning methods such as the standard days method and lactational amenorrhea (LAM). Among women using pills, injectables, IUDs, or implants, less than one-third (29%) were counseled on their method’s side effects and only 37% were told when to return for follow-up. Counseling related to method protection from STIs was even less common. Only 9% of the observed consultations involved a discussion on whether the method protects against STIs, including HIV.

**Table 3:** Family planning counseling by client, provider, and facility characteristics

		Family planning counseling									
		Counseling on how to use method, side effects, and when to return^a^		How to use method^a^		Side effects^b^		When to return^b^		Method protects from STI^a^	
Variable	Category	%	*p*-value	%	*p*-value	%	*p*-value	%	*p*-value	%	*p*-value
Client’s age			0.170		0.115		0.176		**0.005**		0.074
	14–24	22.5		69.1		33.4		47.8		13.1	
	25–39	15.9		59.8		25.7		33.6		8.1	
	40–58 or don’t know	17.1		62.1		31.6		31.9		4.5	
Client’s education			**0.046**		0.092		0.245		0.111		0.288
	No education	13.9		58.4		25.8		32.7		6.8	
	Primary & post primary	22.2		66.5		29.9		40.5		10.8	
	Secondary or more	21.0		67.0		33.6		42.4		11	
Client status			**<0.001**		**<0.001**		**<0.001**		**<0.001**		**<0.001**
	New client	38.1		89.2		53.9		60.8		18.1	
	Returning client	8.9		50.8		18.0		27.0		4.8	
Contraceptive method used			**0.013**		**<0.001**		**0.001**		**<0.001**		0.547
	Pills	15.6		77.0		22.2		26.8		7.1	
	Progestin-only injection	16.0		54.8		27.6		37.4		9.0	
	IUD or implants^c^	29.0		70.3		45.2		54.5		11.4	
Provider category			0.586		0.186		0.592		0.707		0.063
	Nurse or nurse assistant	16.4		58.1		26.9		38.4		13.1	
	Midwife and other	18.5		64.7		29.5		36.5		6.8	
Provider sex			0.955		0.158		0.789		0.257		**0.039**
	Male	18.1		56.3		27.4		42.7		16.1	
	Female	17.8		64.0		29.0		35.9		7.2	
Provider years of education			0.972		0.513		0.401		0.770		0.267
	6–12	18.4		52.9		37.7		31.8		2.3	
	13–16	18.2		64.0		29.8		36.7		10.3	
	17+	17.3		62.0		25.3		38.8		7.7	
Provider training in family planning in the past 24 months			0.644		0.789		0.740		0.394		0.862
	No	17.1		61.9		28.0		35.3		9.1	
	Yes	18.8		63.2		29.5		39.3		8.6	
Provider activities supervised			0.646		0.127		0.277		0.125		0.105
	None	20.4		65.4		34.3		38.2		7.0	
	1–5	15.5		55.9		23.9		43.4		5.3	
	6	18.2		65.3		29.1		33.2		11.7	
Provider has a job description			0.814		0.478		0.803		0.618		0.550
	No	17.3		60.7		28.0		38.4		7.8	
	Yes	18.2		64.0		29.2		36.2		9.6	
Provider salary type			0.346		0.340		0.740		0.532		**0.003**
	Monthly or daily salary	19.3		64.5		29.8		38.6		11.7	
	No regular salary but other compensation	17.1		61.5		27.1		35.1		4.8	
	None	10.7		53.8		25.7		32.8		1.4	
Health facility type			0.138		0.583		0.077		0.120		0.169
	Hospital/health center	13.9		64.4		22.7		31.5		5.9	
	Health post	18.8		62.2		30.1		38.5		9.6	
Locality			0.295		**<0.001**		0.225		0.524		0.263
	Urban	19.7		70.1		31.3		35.8		7.1	
	Rural	15.8		54.4		25.8		38.6		10.7	
Region			0.545		**<0.001**		0.179		**0.010**		0.339
	Northern	11.7		49.1		19.6		29.2		4	
	Dakar	17.2		77.0		30.5		28.0		8.6	
	Thiès	24.3		64.0		40.1		39.0		8.9	
	Central	17.5		44.6		26.8		49.4		8	
	East	24.0		43.1		34.6		26.9		7.3	
	South	18.8		78.6		25.7		48.0		16	
General structure equipment composite index			0.127		0.373		0.093		0.005		0.842
	Low	13.3		64.7		23.1		28.5		7.8	
	Medium	21.8		64.9		34.7		41.6		10	
	High	20.2		58.1		30.7		44.2		8.4	
**Total**		**17.9**		**62.6**		**28.7**		**37.1**		**8.9**	

^a^Applies to all users, only ten respondents reported using methods other than pill, injectable, IUD, or implant

^b^Only applies to users of pill, injectable, IUD, and implant

^c^Includes 10 respondents who used other methods, which were male condoms, LAM, and counseling on periodic abstinence

Note: Bold values indicate a *p*-value<0.05

Associations between counseling and selected characteristics of client, provider, and facility found only a few variations as shown in Table 3. Client status (new or returning) and the contraceptive method they used were significant for almost all counseling items. The client’s age and the education level were not significant for most of the counseling items. Even fewer provider and facility characteristics were found to be significantly associated with the counseling items.

In the adjusted logistic models shown in Table 4, only the client’s status remained significant with new clients having more than six times the odds of receiving counseling compared to returning clients (*p* < 0.001 for both counseling measures). None of the provider or facility characteristics remained significant in the adjusted models except for provider salary type and locality in the counseling on method’s protection from STI. Providers who received a monthly or daily salary had nine times higher odds (*p* < 0.05) of providing counseling on method’s protection from STIs compared with providers without a salary. The only facility characteristic found to be significant was locality and this was only significant for the counseling on method’s protection from STIs. Rural facilities had four times the odds of providing counseling on method’s protection on STIs compared to urban facilities. However, the structure equipment index, which was used as a proxy for the facility’s overall structure, was not significantly associated with the process-dependent variables in the unadjusted and adjusted analysis.

**Table 4:** Adjusted logistic regression of family planning process dependent variables

		Counseled on how to use, side effects, and when to return^a^		Counseled on method protection from STI^b^	
Variable	Category	OR	C.I.	OR	C.I.
Clients age (ref. = 14–24)	25–39	0.7	0.4–1.3	0.7	0.4–1.4
	40–58 & don’t know	1.1	0.6–2.1	0.4*	0.1–0.9
Client’s education (ref. = secondary or more)	No education	0.5	0.3–1.1	0.5	0.2–1.3
	Primary & post primary	1.0	0.5–1.8	1.1	0.4–2.7
Client status (ref. = returning client)	New client	6.1***	**3.7–10.2**	5.3***	**2.8–10.2**
Contraceptive method used (ref. = IUD or implants)	Pills	0.6	0.3–1.4	1.2	0.4–3.4
	Progestin-only injection	0.7	0.4–1.4	1.8	0.8–4.3
Provider category (ref. = midwife and other)	Nurse or nurse assistant	0.8	0.4–1.4	1.4	0.6–3.3
Provider years of education (ref. = 17+)	6–12	1.6	0.4–7.1	0.3	0.1–2.0
	13–16	1.2	0.7–2.2	1.2	0.6–2.7
Provider training in family planning in the past 24 months (ref. = no)	Yes	1.5	0.8–2.7	0.8	0.4–1.9
Provider activities supervised (ref. = none)	1–5	0.6	0.3–1.4	0.8	0.3–2.6
	6	0.7	0.4–1.6	1.4	0.6–3.6
Provider has a job description (ref. = no)	Yes	0.9	0.4–1.8	1.4	0.6–2.9
Provider salary type (ref. = none)	Monthly or daily salary	1.9	0.5–6.4	9.0*	**1.4–58.0**
	No regular salary but other compensation	1.7	0.5–5.5	3.9	0.6–24.5
Health facility type (ref. = hospital/health center)	Health post	1.6	0.9–2.9	1.0	0.4–2.4
Locality (ref. = urban)	Rural	0.8	0.4–1.6	4.0**	**1.4–11.6**
Region (ref. = Northern)	Dakar	1.0	0.2–4.0	2.1	0.5–8.4
	Thiès	1.9	0.6–6.1	2.0	0.4–9.1
	Central	0.9	0.3–3.1	1.1	0.2–5.4
	East	1.6	0.3–7.1	1.0	0.1–9.0
	South	0.8	0.2–2.8	1.8	0.5–7.2
General structure equipment composite index (ref. = low)	Medium	1.8	0.7–4.7	0.5	0.2–1.3
	High	1.9	0.8–4.5	0.5	0.2–1.3
**Pseudo R2**		0.17		0.19	

^a^Only applies to users of pill, injectable, IUD, and implant

^b^Applies to all users, only ten respondents reported using methods other than pill, injectable, IUD, or implant

*p < 0.05, **p < 0.01, ***p < 0.001

Note: Bold values indicate a *p*-value<0.05

### Family Planning Client-Focused Outcomes

For the analysis of the outcome component of quality of care, two outcome variables were examined. The first regression analysis with the outcome of overall satisfaction with family planning services indicates that 84% of users of pills, injectables, IUDs, or implants were very satisfied with the services they received. For the analysis of the second outcome variable, only 58% of the clients had correct knowledge on whether their method protects from STIs (clients using all methods).

Table 5 summarizes the associations of both outcome variables with several covariates representing client, provider, and facility characteristics. The counseling variables produced from the process analysis (from the observation data) were also included as part of the provider variables in the outcome analysis. Waiting time, whether the client left with a method, provider years of education, provider number of activities supervised, having a job description, salary type, region, and the general structure equipment composite index all had significant associations with overall client satisfaction. The greatest difference in satisfaction appears to be by region, as only 59% of clients in the Northern region were very satisfied with services compared with 97% in the South.

**Table 5:** Association of overall client satisfaction with family planning services and client knowledge of family planning method’s protection from STIs by client’s and provider’s background characteristics

		All users that are very satisfied *N* = 872		Correct knowledge of all users *N* = 716	
Variable	Category	(%)	*p*-value	(%)	*p*-value
Client’s age	14–24	82.1	0.312	51.5	0.181
	25–39	83.1		60.3	
	40–58 & don’t know	88.5		59.3	
Client’s education	No education	84.7	0.202	54.5	0.169
	Primary & post primary	85.3		57.6	
	Secondary or more	78.9		64.6	
Fee paid for service	0–9	81.2	0.707		
	10–499	81.7			
	500–999	83.3			
	1000 or more	85.8			
Waiting time	No wait	96.5	**<0.001**		
	Less than half hour	77.0			
	Half hour to one hour	81.1			
	One hour to 2 hours	88.4			
	2 hours or more	83.6			
Client status	New client	84.1	0.859	53.9	0.222
	Returning client	83.5		59.5	
Contraceptive method used	Pills	78.4	0.090	57.6	0.469
	Progestin-only injection	85.3		56.3	
	IUD or implants^b^	86.1		63.9	
Client left with method	Yes	85.1	**<0.001**		
	No	62.3			
Provider category	Nurse or nurse assistant	84.0	0.883	66.6	**0.002**
	Midwife and other	83.6		53.4	
Provider sex	Male	79.5	0.114	61.8	0.300
	Female	84.6		56.7	
Provider years of education	6–12	83.0	**0.007**	61.7	0.700
	13–16	87.1		58.6	
	17+	78.2		55.7	
Provider training in family planning in the past 24 months	Yes	85.3	0.278	63.2	**0.019**
	No	82.4		53.6	
Provider activities supervised	None	73.5	**<0.001**	52.3	**0.004**
	1–5	82.8		49.4	
	6	88.4		64.3	
Provider has a job description	Yes	88.6	**<0.001**	59.8	0.236
	No	76.9		54.9	
Provider salary type	Monthly or daily salary	91.6	**<0.001**	63.0	**0.003**
	No regular salary but other compensation	70.7		49.9	
	None	67.6		46.2	
Counseled on how to use method	Yes	84.4	0.495	60.1	0.137
	No	82.5		53.8	
Counseled on side effects of method^a^	Yes	79.5	0.068	57.4	0.993
	No	85.3		57.5	
Counseled on when to return^a^	Yes	81.6	0.249	57.4	0.983
	No	84.9		57.5	
Counseled on whether method protects from STI	Yes			66.8	0.165
	No			56.7	
Health facility type	Hospital/health center	79.8	0.084	58.8	0.756
	Health post	84.6		57.5	
Locality	Urban	85.0	0.316	57.5	0.922
	Rural	82.3		57.9	
Region	Northern	58.9	**<0.001**	44.9	**<0.001**
	Dakar	87.9		64.6	
	Thiès	75.0		53.4	
	Central	94.5		46.7	
	East	91.5		61.0	
	South	96.7		78.1	
General structure equipment composite index	Low	79.3	**0.020**		
	Medium	85.3			
	High	87.8			

^a^Only applies to users of pill, injectable, IUD, and implant

^b^This includes 10 respondents who use other methods, which were male condoms, LAM, and counseling on periodic abstinence

Note: Bold values indicate a *p*-value<0.05

As Table 6 shows, some of the variables that had significant associations with being very satisfied in the unadjusted models lost their significance in the adjusted logistic regression models. Only client’s waiting time, provider’s years of education, and region remained significant. In addition, client’s education and counseling on side effects, in Model I, and counseling on when to return, in Model II, became significant in the adjusted regression model. The results of the adjusted model indicate that clients who did not receive any counseling had higher odds of being very satisfied compared with clients who received counseling (OR = 2.6, *p* < 0.01 in Model I and OR = 2.0, *p*-value < 0.05 in Model II). In addition, counseling on how to use the method was not significantly related to satisfaction. As for the other variables in the model, the results indicate that clients with lower than secondary education, that did not have to wait to see a provider, and that left with a method were more likely to be very satisfied with services. The results also indicate that clients who were seen by providers having 6–12 years of education or 13–16 years of education had almost three to four times significantly higher odds of being very satisfied compared with clients who saw providers with the highest level of education (17 years or more). The highest odds ratios for being very satisfied were for the region categories, with clients from the South region of Senegal having 12.3 times the odds of being very satisfied compared with Northern Senegal, in Model I (*p* < 0.001), and 13.9 times in Model II (*p*-value < 0.001). Clients in Central Senegal had over 10 times the odds of being very satisfied compared with those in the North region (OR = 10.7, *p* < 0.001, Model I and OR = 11.5, *p* < 0.001, Model II).

**Table 6:** Adjusted logistic regression of clients very satisfied with family planning services and client’s correct knowledge of method’s protection from STIs

Variable	Category	Very satisfied Model I^a^		Very satisfied Model II^a^		Correct knowledge^b^	
		OR	C.I.	OR	C.I.	OR	C.I.
Clients age (ref. = 14–24)	25–39	0.9	0.5–1.6	0.9	0.6–1.6	1.4	0.9–2.2
	40–58 & don’t know	1.9	0.8–4.4	1.8	0.7–4.3	1.3	0.7–2.3
Client’s education (ref. = secondary or more)	No education	2.1*	1.2–3.9	2.1*	1.2–3.8	0.7	0.4–1.2
	Primary & post primary	2.0*	1.0–3.7	2.0*	1.1–3.7	0.9	0.5–1.5
Fee paid for service (ref. = 1000 or more)	0–9	1.3	0.6–3.0	1.4	0.6–3.1		
	10–499	0.9	0.4–2.0	1.0	0.4–2.2		
	500–999	0.9	0.5–1.6	1.0	0.6–1.8		
Waiting time (ref. = 2 hours or more)	No wait	5.4*	1.2–24.0	5.7*	1.3–25.2		
	Less than half hour	0.8	0.4–1.4	0.8	0.4–1.4		
	Half hour to one hour	1.0	0.5–2.1	1.1	0.5–2.2		
	One hour to 2 hours	1.2	0.5–3.0	1.2	0.5–2.8		
Client status (ref. = returning client)	New client	1.2	0.6–2.4	1.1	0.6–2.2	0.7	0.5–1.1
Contraceptive method used (ref. = IUD or implants)	Pills	1.1	0.5–2.7	1.1	0.5–2.6	1.1	0.6–2.0
	Progestin-only injection	1.4	0.7–2.9	1.4	0.6–3.0	0.9	0.5–1.6
Client left with method (ref. = no)	Yes	3.9**	1.5–10.4	3.7**	1.4–10.0		
Provider category (ref. = midwife and other)	Nurse or nurse assistant	1.0	0.6–1.9	1.1	0.6–1.9	2.1**	1.3–3.2
Provider years of education (ref. = 17+)	6–12	3.6*	1.2–10.3	2.9*	1.0–8.3	1.7	0.8–3.7
	13–16	3.8***	2.2–6.6	3.4***	1.9–6.1	1.1	0.8–1.7
Provider training in family planning in the past 24 months (ref. = no)	Yes	1.5	0.9–2.6	1.5	0.9–2.5	1.7**	1.2–2.5
Provider activities supervised (ref. = none)	1–5	1.0	0.6–2.0	1.2	0.7–2.3	0.7	0.4–1.2
	6	0.9	0.5–1.6	0.9	0.5–1.7	1.1	0.7–1.9
Provider has a job description (ref. = no)	Yes	1.1	0.6–1.9	1.1	0.6–1.9	1.0	0.6–1.5
Provider salary type (ref. = none)	Monthly or daily salary	2.0	0.9–4.5	2.0	0.9–4.6	1.3	0.7–2.5
	No regular salary but other compensation	0.9	0.4–1.8	0.9	0.4–1.8	1.3	0.7–2.5
Counseled on how to use method (ref. = no)	Yes	1.3	0.8–2.2	1.3	0.8–2.2		
Counseled on side effects of method^a^ (ref. = yes)	No	2.6**	1.5–4.6				
Counseled on when to return^a^ (ref. = yes)	No			2.0*	1.2–3.5		
Counseled on whether method protects from STI (ref. = no)	Yes					1.2	0.6–2.3
Health facility type (ref. = hospital/health center)	Health post	1.5	0.8–2.8	1.4	0.8–2.6	0.7	0.5–1.1
Locality (ref. = urban)	Rural	0.9	0.5–1.7	1.0	0.5–1.8	1.3	0.8–2.0
Region (ref. = Northern)	Dakar	5.3***	2.1–12.9	4.8***	1.9–11.9	3.0**	1.4–6.3
	Thiès	2.8*	1.2–6.6	2.5*	1.1–5.9	1.9	1.0–3.7
	Central	10.7***	3.6–32.3	11.5***	3.7–36.3	1.3	0.6–2.6
	East	5.2*	1.4–19.0	4.6*	1.3–16.6	2.0	0.8–4.9
	South	12.3***	3.4–45.4	13.9***	3.7–52.5	4.2***	1.9–9.2
General structure equipment composite index (ref. = low)	Medium	0.9	0.5–1.7	0.9	0.5–1.5		
	High	1.0	0.5–2.0	1.0	0.5–1.9		
**Pseudo-R** ^**b**^		0.25		0.25		0.09	

For client satisfaction outcome, Model I includes the variable for counseled on side effects, and

Model II includes the variable on counseled on when to return. ^a^Users of pill, injectable, IUD, and implant. ^b^All users

*p < 0.05, **p < 0.01, ***p < 0.001

Note: Bold values indicate a *p*-value<0.05

As Table 5 shows, it was mainly the provider’s characteristics that had significant associations with the client’s knowledge of whether their method protects from STIs. None of the client’s background characteristics were significantly associated with their knowledge. Only three independent variables remained significant in the adjusted logistic regression model: provider category, provider family planning training, and region. Clients who saw a nurse or nurse’s assistant had twice the odds of having correct knowledge compared with clients who saw a midwife or other provider (OR = 2.1, *p* < 0.01). Similarly, clients who saw a provider who received family planning training in the last two years had higher odds of having correct knowledge compared with clients who saw a provider with no recent training (OR = 1.7, *p* < 0.01). However, receiving counseling from a provider on the method’s protection from STIs was not a significant predictor of having correct knowledge in this area. For region, only the South and Dakar had significant odds ratios in the logistic regression model for correct knowledge. Clients in the South had 4.2 times the odds of having correct knowledge compared with clients in the North (*p* < 0.001), while clients in Dakar had three times the odds of having correct knowledge compared with Northern Senegal (*p* < 0.01).

## Discussion

The results indicate that provider counseling during client visits to family planning services in Senegal may not be adequate or effective. Especially low levels of counseling were observed in provider counseling on method’s protection from STIS, with only 9% of providers observed to have given this counseling. Only 18% of providers were observed to give counseling on all three important aspects of the method they are using: how to use the method, possible side effects, and when to return to the facility. The adjusted logistic regression of these two counseling outcomes have shown that new clients had a higher odds of receiving counseling compared to returning clients. Perhaps providers assumed that returning clients had already been counseled about their method in previous visits and thus did not need further counseling at each visit. However, counseling may be required more than once to ensure that clients understand fully how to use their family planning method, are aware of the side effects, and know whether their method protects from STIs. Only 9% of the providers were observed to provide counseling on whether the client’s method protects from STIs, and 42%of clients had incorrect knowledge of whether their family planning method protects from STIs-- many women believe incorrectly that their method protects them from STIs.

There were no differences between the provider’s characteristics in terms of counseling on how to use method, side effects and when to return. In addition, only provider’s salary was shown to be significant for counseling on method’s protection from STIs: providers that have a salary have 9 times the odds of providing this counseling compared to providers with no salary. This difference may indicate that providers with no salary are less motivated or less inclined to provide counseling. Rowe et al. [24] mentioned that the administrative environment of health workers, including salary, could influence their performance. Another important provider factor associated with the quality of services is personal supervision received by the provider [25]. Supervision, especially with resulting feedback, can directly influence quality of care. After a formative supervision intervention in health facilities in four districts of Senegal, significant improvement was observed in a range of service areas across all districts [26]. However, in the present analysis, supervision did not have an effect on whether the provider gave counseling in the adjusted models. This may be an indication of the quality of the supervision given to the providers but this needs further study.

Interestingly, providers working in rural facilities had a higher odds of providing counseling on method’s protection of STIs compared to urban facilities. However, the equipment composite index which was used as a proxy for the facility’s structure was not found to be a significant predictor of counselling. In addition, virtually no significant relationships were found between the basic infrastructure indicators (such as availability of electricity, improved water source, adequate sanitation facilities, etc.) and the process-dependent variables (results not shown). The link between structure and process has been reported as being weak by Donabedian [16]; and Basinski et al. [27] indicated that this relationship depends on which structure and process components are compared. Because providing the various forms of counseling does not differ greatly by the client, provider, or facility characteristics (with some exceptions) it appears that the promotion of adequate counseling is required in all the health facilities of Senegal.

Analysis of the outcomes of family planning visits (satisfaction with services and knowledge of method’s protection from STIs) has shown that improvements are required in the quality of counseling provided to clients. Effective counseling can improve outcomes; a study on client-centered versus physician-centered consultations found that clients who received a client-centered consultation significantly increased their likelihood of satisfaction with services and method continuation at seven months [13]. In the present analysis, counseling on side effects and when to return showed a negative effect on satisfaction and counseling on how to use a method had no effect on satisfaction. In addition, counseling on method’s protection from STIs had no effect on improving knowledge in this area. These findings perhaps imply that the provider/client interaction during counseling was unsatisfactory. In a study in Zambia, the odds of correct knowledge were higher among clients who were counseled in their method’s protection from STIs [14]. That study also found a much higher proportion of clients who had correct knowledge compared with Senegal—75%—among users of a method other than condoms. The apparent lack of impact of the counseling on STIs in Senegal suggests that this type of counseling offered in the health facilities may be of poor quality. This result is similar to the absence of a significant impact of counseling on how to use a method and a *negative* impact of counseling on side effects, and when to return, on client satisfaction with family planning services. In addition, it could be that the topic of counseling on side effects and when to return is discouraging to clients as perhaps they are told of the problems they may face by using the method. Further analysis on the effectiveness and quality of the counseling provided in family planning services may be required to understand why counseling is not improving outcomes.

The apparent lack of effective counseling could be a result of the manner in which the counseling was provided, including the provider/client interpersonal relationship, which is an important aspect of providing quality of care in family planning (Bruce 1990) and maternal health care (Srivastava et al. 2015). The results have shown that clients who have seen a provider with less than 17 years of education had higher odds of being very satisfied and having correct knowledge compared to clients who have seen a provider with 17 years or more of education. This was also found in a study on quality of care in family planning clinics in Iran [28]. This finding could be an indication of the disconnect between the client and the provider and the poor provider/client interpersonal relationship that appears to be higher with the most educated providers.

Although most of the health facilities analyzed had at least one staff member trained in family planning (95%), only 45% of providers who prescribed or provided a modern family planning method had receiving family planning training in the past two years (Table 2). The differences in family planning training by provider category were not significant (results not shown). These findings imply that training providers in family planning could improve the client’s knowledge of their method’s protection from STIs. While correct knowledge could be gained elsewhere and not only from a family planning provider, providing more training for midwives and other types of providers, as well as providing more facilities with family planning guidelines, may be effective interventions to improve client knowledge of STI protection.

In the 2015 Senegal SPA, observation and exit interviews will be available for family planning, and future analysis can provide comparisons between the 2012–2013 and 2015 rounds of the Senegal Continuous SPA surveys. This type of analysis may provide insight as to whether improvements have been made in the process and outcome measures of quality of care in family planning services and whether counseling is able to improve outcomes.

### Limitations

The complexity of defining quality of care, as well as selecting and constructing the indicators, is one of the limitations of the study. For the outcome measures selected, client satisfaction may be over-reported due to the client perhaps not wanting to speak against their providers [16]; satisfaction is also subjective. Knowledge of whether a family planning method protects from STIs could be gained from other sources and may not be attributed to the providers or facility characteristics; we also do not know the level of knowledge of the client on the subject before the family planning visit. In addition, the counseling measures used were simply binary variables of whether the provider gave the counseling or not and does not include information on the quality of the counselling. It can be difficult to find appropriate and objective outcome measures of quality of care, since health outcomes do not depend solely on the quality of care. Finally, there is also the question of whether providers who know they are being observed are providing better or more counseling than normal. That is, they may perform differently under observation, a phenomenon known as the Hawthorne Effect [29, 30]. Even though the overall percentage of providers offering different types of counseling is relatively low, the percentage could be even lower when the providers are not being observed.

## Conclusions

Improvements may be required in the level and effectiveness of counseling provided by health workers. The percentage of providers who gave the necessary family planning counseling to their clients was relatively low. New clients were more likely to receive counseling compared with returning clients. Few characteristics of clients, providers, and facilities were significant predictors of receiving counseling. The effectiveness of the counseling provided in facilities with family planning services was not seen in the analysis of the outcomes of overall satisfaction and the client’s knowledge of their method’s protection from STIs. Clients who were counseled on side effects and when to return were less likely to be very satisfied, and whether clients received counseling on their method’s protection from STIs was not a significant predictor of having correct knowledge. This may indicate that not only is more counseling required in the health facilities in Senegal, but also more training may be required on how to provide more effective and client-centered family planning counseling. Clients who were seen by a provider with family planning training were almost twice as likely to have correct knowledge compared with clients who saw a provider with no training. This indicates that more training in family planning may be another desirable type of intervention for improving quality of family planning services.

## Abbreviations

FP: Family planning
IUD: Intrauterine device
SPA: Senegal service provision assessment
